# The Syphilis Epidemic Among Heterosexuals Is Accelerating: Evidence From King County, Washington

**DOI:** 10.1093/ofid/ofad481

**Published:** 2023-09-23

**Authors:** Anna Berzkalns, Meena S Ramchandani, Chase A Cannon, Roxanne P Kerani, Julie C Dombrowski, Matthew R Golden

**Affiliations:** HIV/STD/HCV Program, Public Health–Seattle & King County, Seattle, Washington, USA; HIV/STD/HCV Program, Public Health–Seattle & King County, Seattle, Washington, USA; Department of Medicine, University of Washington, Seattle, Washington, USA; HIV/STD/HCV Program, Public Health–Seattle & King County, Seattle, Washington, USA; Department of Medicine, University of Washington, Seattle, Washington, USA; HIV/STD/HCV Program, Public Health–Seattle & King County, Seattle, Washington, USA; Department of Medicine, University of Washington, Seattle, Washington, USA; Department of Epidemiology, University of Washington, Seattle, Washington, USA; HIV/STD/HCV Program, Public Health–Seattle & King County, Seattle, Washington, USA; Department of Medicine, University of Washington, Seattle, Washington, USA; Department of Epidemiology, University of Washington, Seattle, Washington, USA; HIV/STD/HCV Program, Public Health–Seattle & King County, Seattle, Washington, USA; Department of Medicine, University of Washington, Seattle, Washington, USA; Department of Epidemiology, University of Washington, Seattle, Washington, USA

## Abstract

**Background:**

We characterized the rapid increase in syphilis among cisgender women in King County, Washington, and compared it with trends among cisgender men who have sex with men.

**Method:**

We used surveillance data from King County, 2007 to 2022, to describe incidence trends stratified by syphilis stage, gender, and gender of sex partners; trends in pregnant cases and congenital syphilis; and trends in rapid plasma reagin titer at diagnosis among late/unknown duration cases. We used joinpoint regression to analyze trends.

**Results:**

Among cisgender women, all-stage syphilis incidence remained stable from 2007 to 2010 but then increased by 16.3% per year (95% CI, 12.0%–20.7%) from 2010 to 2020 and 90.1% per year (95% CI, 26.4%–185.9%) from 2020 to 2022. Early syphilis rates rose gradually from 2007 to 2017 (18% per year; 95% CI, 7.4%–29.6%) and then rapidly from 2017 to 2022 (62.5% per year; 95% CI, 24.1%–112.9%). In contrast, the increase in late/unknown duration syphilis incidence was delayed. Among cisgender men who have sex with women, all-stage syphilis remained stable from 2007 to 2014 and increased 25.0% per year (95% CI, 14.0%–37.0%) from 2014 to 2022. Syphilis incidence increased steadily among men who have sex with men, with all-stage incidence increasing 7.0% per year (95% CI, 4.8%–9.2%) from 2007 to 2022. Median rapid plasma reagin titer among late/unknown duration cases increased significantly over the analysis period.

**Conclusions:**

An explosive epidemic of syphilis is ongoing in King County. The delayed increase in asymptomatic late/unknown duration cases relative to early symptomatic cases suggests that there is a large and growing reservoir of recently acquired undiagnosed syphilis in women. New clinical and public health activities are urgently needed to control the growing epidemic.

## Teaser

This joinpoint regression trend analysis compared syphilis incidence in King County, Washington, among cisgender women and cisgender men who have sex with men. Results suggest that there is a large and growing reservoir of recently acquired, undiagnosed syphilis in women.

The United States is in the midst of the largest syphilis epidemic among women in a quarter century with national rates of primary and secondary syphilis increasing >7-fold between 2013 and 2021 [1]. The last major syphilis epidemic in US women occurred in the late 1980s and early 1990s [2, 3]. Since then, syphilis control has concentrated primarily on the epidemic among men who have sex with men (MSM), with epidemiologic monitoring of syphilis generally focused on the number of cases of early-stage infection (primary, secondary, and early nonprimary nonsecondary [formerly early latent]). This approach reflects a desire to concentrate surveillance efforts and resource-intensive public health partner services on cases that were recently acquired. Such priority cases often include those with a high rapid plasma reagin (RPR) titer, which are usually early stage and thought to be more infectious.

The recent epidemic of syphilis in heterosexuals presents a different epidemiologic challenge. While many MSM frequently test for sexually transmitted infections (STIs), allowing for the identification of asymptomatic recently acquired infections, relatively few heterosexuals regularly test for syphilis. As a result, it is often not possible to determine when a woman or heterosexual man with asymptomatic syphilis may have been infected. In the absence of symptoms, a confirmed contact to an early syphilis case, or a negative syphilis test result in the past year, clinicians and public health authorities stage syphilis cases as unknown duration. Cases of late syphilis, including infections of unknown duration, typically have lower RPR titers than early latent cases, but there is substantial overlap in the distribution of titers between early and late/unknown cases, suggesting that many cases categorized as unknown duration were recently acquired [4].

Here we report on the rapid increase in syphilis among cisgender women in King County, Washington, and contrast this recent epidemic to our longer-standing epidemic in cisgender MSM. Our analysis highlights how monitoring trends in early and late/unknown duration syphilis provides a better sense of the true scale of the epidemic in cisgender women and suggests that there is an underappreciated but growing reservoir of asymptomatic infections.

## METHODS

We used King County STI surveillance data to obtain syphilis case counts from 2007 to 2022. The STI surveillance system data include syphilis stage, sex assigned at birth, gender, diagnosis date, gender of sex partners, pregnancy status, and laboratory test results. We calculated syphilis incidence from 2007 to 2022 among cisgender women, cisgender MSM, and cisgender men who have sex with women (MSW). Information on the gender of sex partners was obtained from provider report and patient interview. Case gender was defined by using a combined gender/sex field from 2007 to February 2020 (single-question period) and separate sex assigned at birth and current gender fields from March 2020 to 2022 (2-question period). Cases were defined as cisgender during the single-question period if no transgender identity was reported and during the 2-question period if the current gender matched the sex assigned at birth. Male cases were defined as MSM if the patient or provider reported male sex partners in the past year and MSW if the patient or provider reported only female sex partners in the past year. We chose 2007 as the initial year for the study to limit analyses to data stored in a single surveillance system that included relatively complete data on the gender of sex partners and RPR titer at diagnosis.

We calculated incidence rates using population estimates provided by the Washington State Office of Financial Management for intercensal years and US census annual population estimates for 2020. For 2021 and 2022, preliminary population counts were used. The MSM population estimate comes from data in the King County–specific Behavioral Risk Factor Surveillance System (BRFSS) [5, 6]. For 2013 and earlier, we assumed that 5.7% of men aged ≥15 years in King County were MSM based on BRFSS data collected in 2013 and 2014. From 2014 onward, the size of the MSM population was estimated with the 2-year average of the percentage of men who reported being gay or bisexual in BRFSS by using data from the 2 years prior to the year for which STI incidence was estimated. This is the standard approach employed by our health department for HIV/STI surveillance monitoring. The percentage of men aged ≥15 years estimated to be MSM from 2014 to 2022 is as follows: 2014 (6.2%), 2015 (6.3%), 2016 (6.4%), 2017 (6.6%), 2018 (6.7%), 2019 (6.5%), 2020 (6.4%), 2021 (6.5%), and 2022 (6.5%).

We compared the incidence of early and late/unknown duration syphilis and the proportion of all syphilis cases per year in each staging category. We analyzed trends in overall, early, and late/unknown duration syphilis among cisgender women, MSW, and MSM using joinpoint regression [7], a statistical approach to test for statistically significant changes in trend. An additional analysis separately evaluated trends in primary and secondary syphilis (combined) and early nonprimary nonsecondary cases, trends among cisgender women who were pregnant at diagnosis, and trends in congenital syphilis. We also compared trends in RPR titer at diagnosis among late/unknown duration cases for the following periods: 2007 to 2010, 2011 to 2020, and 2021 to 2022. These periods were chosen from the results of the cisgender women all-stage syphilis joinpoint regression. Trend analyses evaluated changes in the proportion of cases in each period with an RPR titer ≥1:32 via the Cochran-Armitage test for trend and compared median RPR titers per the Kruskal-Wallis test. Joinpoint regression analysis was based on the Joinpoint Regression Program version 4.9.1.0 [8]. SAS version 9.4 (SAS Institute) was used for all other analyses.

### Patient Consent Statement

This analysis was conducted as part of routine public health surveillance activities and exempt from Institutional Review Board review.

## RESULTS

### Syphilis Trends in Cisgender Women

A total of 1215 all-stage syphilis cases were diagnosed among cisgender women residing in King County from 2007 to 2022 (Table 1): 33% were White, 22% Black, and 13% Hispanic/Latinx; most were 24 to 44 years of age. Of the 1215 cisgender women with syphilis, 567 (47%) were interviewed for the purposes of partner services, among whom 20% were living homeless, 7% reported injection drug use, 22% methamphetamine use, and 6% exchange sex. A specific question about homelessness was added to interviews in 2020, and since that time 30% of cisgender women interviewed following a syphilis diagnosis reported being homeless.

**Table 1.** Characteristics of All-Stage Syphilis Cases Among Cisgender Women, King County, Washington, 2007–2022

Characteristic	No.	%
Total cases	1215	100
Race/ethnicity		
American Indian/Alaska Native	29	2
Asian	95	8
Black	271	22
Hispanic/Latinx	161	13
Native Hawaiian or Pacific Islander	28	2
White	395	33
Multiracial	44	4
Other/unknown	192	16
Age, y		
10–14	3	0
15–19	51	4
20–24	167	14
25–29	206	17
30–34	208	17
35–44	290	24
45–54	172	14
≥55	118	10
Total interviewed	567	47
Risk factors among interviewed cases ^a^		
Homeless/unstable housing, past 3 mo	116	20
Past year		
Injection drug use	42	7
Methamphetamine use	122	22
Exchange sex	34	6

^a^Risk factor percentages among 567 interviewed cases.

**Figure 1.**
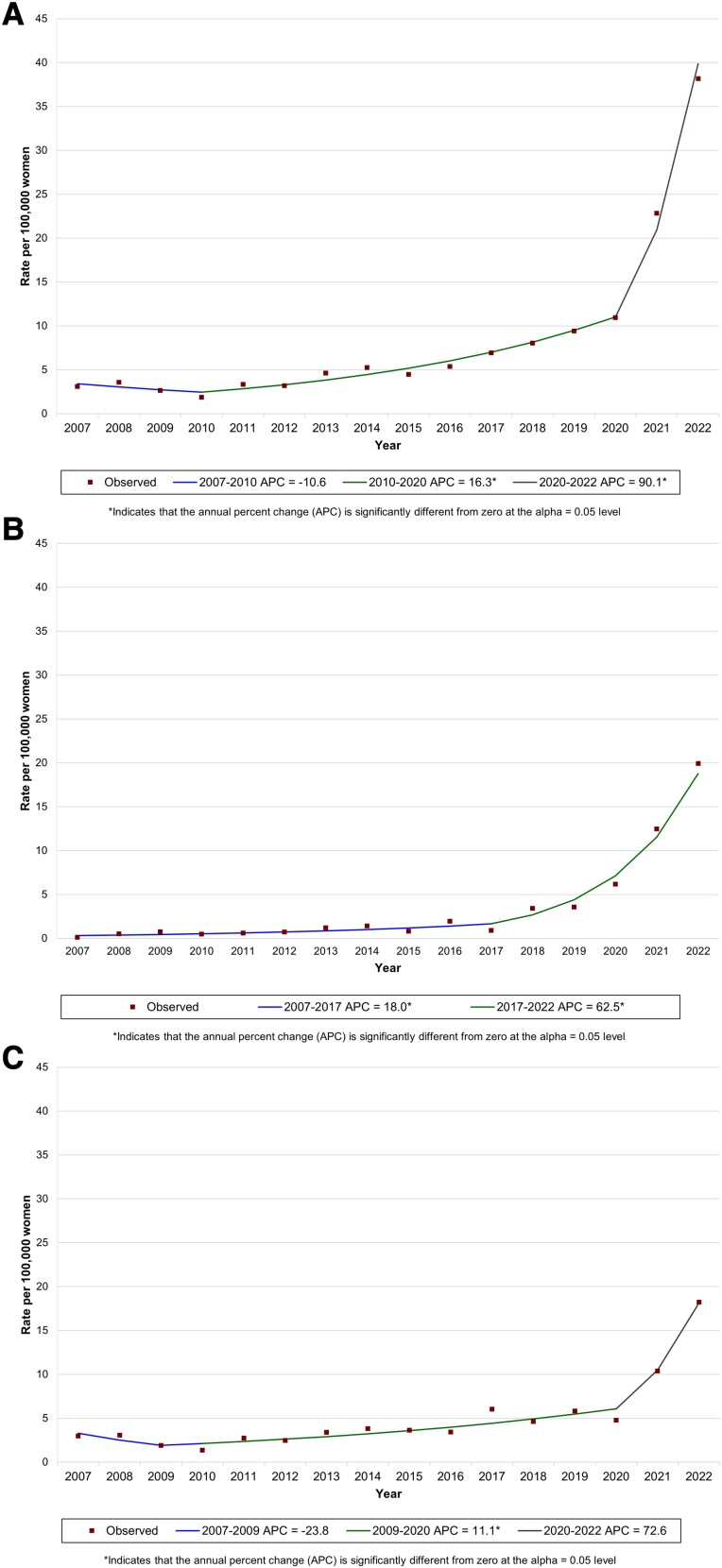
Joinpoint regression analysis of trends in the rate of syphilis among cisgender women in King County, Washington, 2007–2022: *A*, all stages; *B*, early; *C*, late/unknown duration.

**Figure 2.**
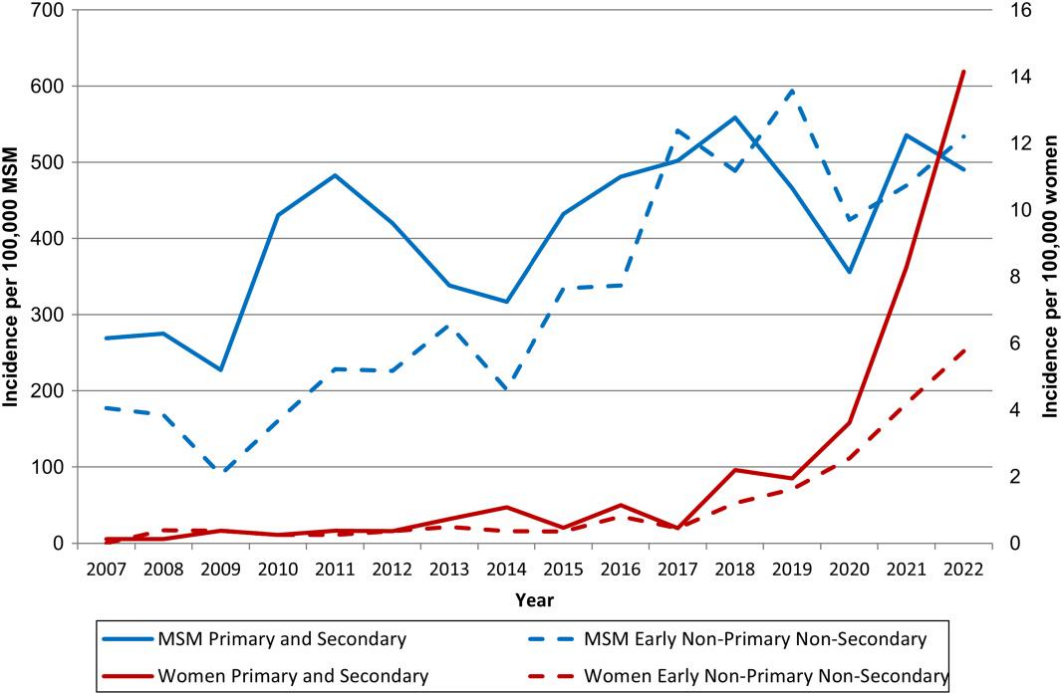
Early syphilis incidence comparing primary and secondary syphilis with early nonprimary nonsecondary syphilis among cisgender women and men who have sex with men (MSM), King County, Washington, 2007–2022.

Joinpoint analysis defined 3 phases in all-stage syphilis incidence among King County cisgender women between 2007 and 2022 (Figure 1*A*, Table 2). The incidence of all-stage syphilis remained roughly stable from 2007 to 2010, increased by an average of 16.3% per year (95% CI, 12.0%–20.7%) from 2010 to 2020, and then rose dramatically by 90.1% per year (95% CI, 26.4%–185.9%) from 2020 to 2022. However, this pattern of increase obscures the heterogeneity evident in joinpoint analyses that separately evaluated trends in early and late/unknown duration syphilis. Early syphilis incidence rose relatively gradually from 2007 to 2017 (18.0% per year; 95% CI, 7.4%–29.6%) and then rapidly between 2017 and 2022 (62.5% per year; 95% CI, 24.1%–112.9%; Figure 1*B*). Within this trend in early syphilis in cisgender women, primary/secondary and early nonprimary nonsecondary syphilis increased, but the magnitude of that increase varied. The incidence of primary/secondary syphilis increased from 0.1 to 14.2 per 100 000, while the incidence of nonprimary nonsecondary syphilis increased by a much smaller amount, from 0 to 5.8 per 100 000 (Figure 2). This divergence in trends was pronounced from 2020 to 2022.

**Table 2.** Trends of Syphilis Incidence Overall and by Syphilis Stage Among Cisgender Women, Cisgender MSM, and Cisgender MSW by Joinpoint Analysis, King County, Washington, 2007–2022

Population and Syphilis Stage: No. of Joinpoints	APC (95% CI)	*P* Value
Cisgender women		
All stages: 2		
2007–2010	−10.6 (−27.1 to 9.6)	.239
2010–2020	16.3 (12.0 to 20.7)	<.001
2020–2022	90.1 (26.4 to 185.9)	.007
Early: 1 ^a^		
2007–2017	18.0 (7.4 to 29.6)	.003
2017–2022	62.5 (24.1 to 112.9)	.002
Late/unknown duration: 2		
2007–2009	−23.8 (−61.4 to 50.4)	.383
2009–2020	11.1 (5.4 to 17.2)	.002
2020–2022	72.6 (−12.6 to 240.8)	.101
Cisgender MSM		
All stages: 0 (2007–2022)	7.0 (4.8 to 9.2)	<.001
Early: 0 (2007–2022)	6.8 (4.4 to 9.2)	<.001
Late/unknown duration: 0 (2007–2022)	8.2 (6.0 to 10.5)	<.001
Cisgender MSW		
All stages: 1		
2007–2014	1.2 (−9.5 to 13.3)	.814
2014–2022	25.0 (14.0 to 37.0)	<.001
Early: 0 (2007–2022)	21.5 (14.1 to 29.4)	<.001
Late/unknown duration: 0 (2007–2022)	7.3 (2.2 to 12.7)	.008

Abbreviations: APC, annual percentage change; MSM, men who have sex with men; MSW, men who have sex with women.

^a^Primary, secondary, early nonprimary nonsecondary.

In contrast to the pattern seen in early syphilis, joinpoint analyses for trends in late/unknown duration syphilis among cisgender women defined 3 phases: a period of small nonsignificant decline from 2007 to 2009, a period of gradual increase from 2009 to 2020 (11.1% per year; 95% CI, 5.4%–17.2%), and a period of accelerated epidemic growth from 2020 to 2022 (72.6% per year; 95% CI, −12.6% to 240.8%; Figure 1*C*). Thus, the abrupt increase in late/unknown syphilis was delayed, starting approximately 3 years after the increase in early syphilis began to accelerate.

The differential trends in syphilis by stage among cisgender women resulted in a shift in the relative incidence of early vs late/unknown duration syphilis. From 2007 to 2019, most (76%) syphilis cases in women each year were classified as late/unknown duration (range, 58%–96%). As the rate of early syphilis climbed, the percentage of syphilis cases in cisgender women classified as early also increased. By 2020, the rate of early syphilis among women exceeded that of latent/unknown duration syphilis, and in 2022, 52% of all syphilis cases in women were early.

### Trends in Syphilis Among Pregnant Cisgender Women and Congenital Syphilis

The increase in syphilis among cisgender women was associated with a rise in the number of cases diagnosed in pregnancy (Supplementary Figure 1). The number of pregnant syphilis cases was stable at a median 1 per year between 2007 and 2014, though pregnancy status was not added to the case report until October 2014, possibly resulting in an underestimate. From 2016 to 2020, Public Health–Seattle & King County (PHSKC) identified a median 14 pregnant cases per year. This increased dramatically in 2021 to 2022, with 32 cases of syphilis occurring in pregnant women in 2022, 81% of which were classified as late/unknown duration. The number of congenital syphilis cases increased concurrent with the increase in syphilis among women, with 11 cases occurring in 2021 and 12 in 2022.

### Trends in Syphilis Among Cisgender MSW and MSM

The incidence of all-stage syphilis among King County MSW increased 703% between 2007 and 2022 and mirrored the trends observed in women (Supplementary Figure 2). Between 2007 and 2022, joinpoint analysis defined 2 phases in all-stage syphilis incidence among MSW: syphilis remained stable from 2007 to 2014 and increased by an average of 25.0% per year (95% CI, 14.0%–37.0%) from 2014 to 2022 (Supplementary Figure 3, Table 2). Similar to the increases among cisgender women, larger increases in early syphilis as compared with late/unknown duration syphilis were observed among MSW. Early syphilis and late/unknown syphilis increased over the study period by an average per year of 21.5% (95% CI, 14.1%–29.4%) and 7.3% (95% CI, 2.2%–12.7%), respectively, with no points of change identified by joinpoint analysis. Syphilis rates among King County MSM also rose substantially between 2007 and 2022. However, the relative magnitude of that increase was much smaller when compared with MSW; its progression was sporadic; and the pattern of increase by syphilis stage was different (Supplementary Figure 4). Joinpoint analysis did not identify any points at which incidence among MSM changed (Table 2); incidence increased steadily by an average of 7.0% (95% CI, 4.8%–9.2%) a year between 2007 and 2022 (Supplementary Figure 5). Early syphilis and late/unknown syphilis increased over this period by an average per year of 6.8% (95% CI, 4.4%–9.2%) and 8.2% (95% CI, 6.0%–10.5%). In contrast to the trend observed among cisgender women, early syphilis among MSM was substantially more common than late/unknown duration syphilis throughout the study period, with a median 84% of annual cases being classified as early over the 16-year period (range, 78%–89%).

### Trends in RPR Titer

**Figure 3.**
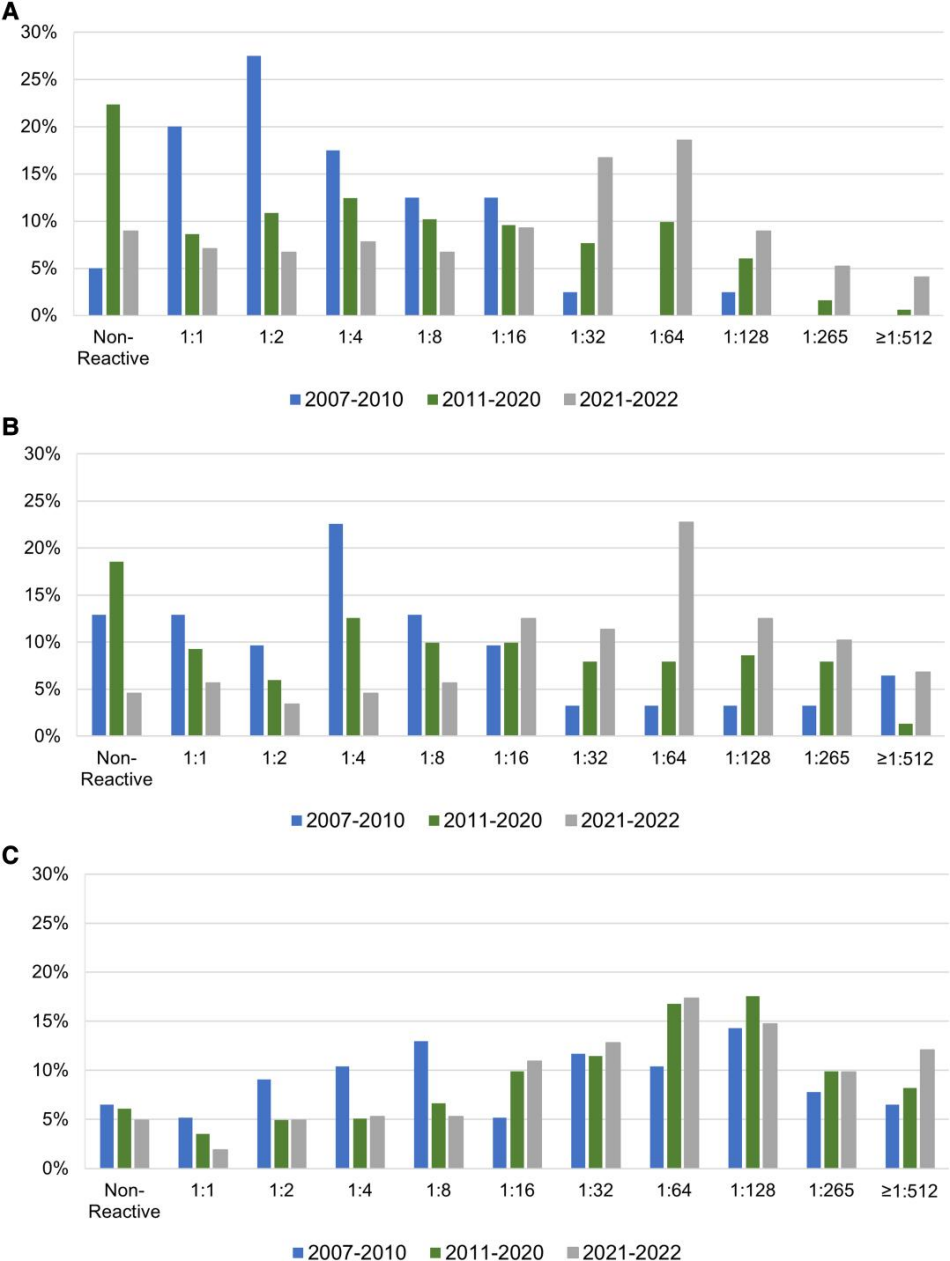
Trends in rapid plasma reagin titer with late/unknown duration syphilis, King County, Washington, 2007–2022: *A*, cisgender women; *B*, cisgender men who have sex with women; *C*, cisgender men who have sex with men.

The increase in the rate of late/unknown duration syphilis observed in cisgender women was characterized by a shift in the distribution of RPR titers (Figure 3*A*). The median RPR titer among cisgender women with late/unknown duration syphilis in 2007 to 2010, 2011 to 2020, and 2021 to 2022 was 1:2, 1:4, and 1:32, respectively (*P* <.0001), while the percentage of cases with an RPR titer ≥1:32 was 5%, 26%, and 54% (*P* < .0001). The same pattern of increasing RPR titer occurred among cisgender MSW diagnosed with late/unknown duration syphilis (Figure 3*B*), with the median titer increasing from 1:4 in 2007 to 2010 to 1:8 in 2011 to 2020 to 1:64 in 2021 to 2022 (*P* < .0001); the percentage of cases with an RPR titer ≥1:32 was 19%, 34%, and 64% (*P* < .0001). While RPR titers among cisgender MSM with late/unknown duration syphilis also increased, most late/unknown duration cases among MSM had titers >1:32 throughout the period, and the increase in titer was smaller than that observed in cisgender women and MSW (Figure 3*C*). The median titer among MSM in 2007 to 2010, 2011 to 2020, and 2021 to 2022 was 1:32, 1:64, and 1:64 (*P* = .0249), and the percentage of cisgender MSM with late/unknown duration syphilis cases with an RPR titer ≥1:32 was 51%, 64%, and 67% (*P* = .0302).

## DISCUSSION

Analyzing data from King County from 2007 to 2022, we characterized an accelerating syphilis epidemic among cisgender women and MSW. The rising epidemic among women was associated with increasing numbers of cases among pregnant persons and congenital syphilis cases and displayed an epidemiologic pattern that was distinct from the long-standing, gradual rise in syphilis rates among MSM. The current epidemic started in 2017 and was initially characterized by an increase in early syphilis, most of which was diagnosed as symptomatic primary and secondary infections. This was followed by a delayed, very rapid increase in asymptomatic late/unknown duration infections starting in 2020. Most late/unknown duration cases had high RPR titers consistent with recently acquired infection. Our findings suggest that there is a large and growing reservoir of recently acquired and potentially infectious, undiagnosed syphilis in cisgender women.

King County is not exceptional in its experience. Nationally, the rate of all-stage syphilis among women aged 15 to 44 years increased 36% between 2020 and 2021. The Western United States has been particularly affected by the rise in syphilis among women. Among the 13 states in the region, the syphilis rate among women aged 15 to 44 years increased by a median 1900% between 2012 and 2021 vs 933%, 288%, and 475% in states in the Midwest, South, and Northeast, respectively. The median rate of syphilis among women aged 15 to 44 years in Western states now exceeds that of any other region [1]. Our surveillance data from King County, which include more recent data than are available nationally and specific information on cases in MSM and MSW, provide a more nuanced picture of an evolving epidemic and suggest several conclusions and areas for urgent action.

First, our findings suggest that the scale of the current epidemic among cisgender women and MSW is likely substantially larger than previously indicated by surveillance data that rely on case reporting. Traditionally, most cases of syphilis diagnosed among cisgender women in King County were classified as being late/unknown duration, and most late/unknown duration cases had low RPR titers. This pattern reflected a low rate of syphilis transmission among heterosexuals resulting in few symptomatic cisgender women (ie, small numbers of primary and secondary syphilis cases) and relatively few late/unknown duration cases with high RPR titers. Most diagnoses occurred in cisgender women who had long-standing, low-titer syphilis, likely reflecting infections with low transmissibility that had accumulated in the population over many years. That is no longer the case. Most cases are now symptomatic, and a rapidly growing number of asymptomatic cases have high RPR titers. We believe that the best interpretation of our data is that we initially saw a rise in symptomatic cases but these represented a fraction—perhaps a minority—of all new infections. Screening of nonpregnant women and MSW was relatively uncommon, leading to a growing reservoir of recently acquired, high-titer infections. With little to no increase in screening, the identification of large numbers of these asymptomatic cases was delayed until prevalence grew substantially, leading to a delayed surge in the identification of asymptomatic high-titer infections.

Other interpretations of our epidemiologic findings are possible. The number of identified infections could increase if there was a large increase in syphilis testing in the at-risk population. However, the number of RPR syphilis tests performed by Labcorp—a large private reference laboratory—on specimens provided by women in Washington State increased only 34% between 2019 and 2022 (Samia Naccache, personal communication), a period during which syphilis cases among women increased >300%. It is also possible that most infections in heterosexuals are being diagnosed while patients are still symptomatic; as such, we believe that it is more likely that the reservoir of undiagnosed asymptomatic infection is rapidly growing. Among MSM—a population that includes many men who are familiar with the symptoms of syphilis and see medical providers experienced with diagnosing syphilis—less than half of cases are diagnosed because of symptoms [9]. While some missed early infections among MSM may be attributable to attenuated symptoms in the setting of prior immunologic experience with *Treponema pallidum* [10], we think that it is unlikely that early diagnosis is more common in heterosexuals than MSM given (1) the heterosexual population's lower familiarity with the clinical presentation of syphilis, (2) the variable experience of their medical providers, and (3) historical experience suggesting that early infections go undiagnosed more often in women than men [11, 12]. The conclusion that we are likely failing to diagnose large numbers of women while they are symptomatic—and that the reservoir of undiagnosed infection is substantial—is also consistent with the high prevalence of syphilis (3%–6.5%) recently observed among women and MSW in jails in King County and Los Angeles [13] (personal communication, Magdelena Esquivel).

Second, our findings highlight the need to expand the epidemiologic monitoring of syphilis to include all cases, as well as late/unknown duration infection. The Centers for Disease Control and Prevention recently began including tables on all-stage syphilis in the national sexually transmitted disease surveillance report [1]. State and local jurisdictions should likewise expand their surveillance activities to monitor all asymptomatic infections, particularly high-titer late/unknown duration infections. Persons diagnosed with syphilis with no known contact with an early case, no negative test result in the prior year, and no signs or recent symptoms are categorized as having infections of unknown duration. Insofar as surveillance monitoring neglects these cases, the result is a substantial underestimate of the true size of the epidemic.

Finally, our observations should prompt additional clinical and public health activities to control the growing epidemic. The Washington State Department of Health and PHSKC recently released expanded syphilis screening guidelines that recommend frequent testing of populations at high risk for syphilis (ie, persons who are unstably housed, people who use opioids or methamphetamine, persons engaged in exchange sex, MSM, and transwomen who have sex with men), as well as testing of all sexually active persons aged ≤45 years who have not tested for syphilis since January 2021 [14]. These guidelines also recommend expanded screening for pregnant persons. Health departments in California and Oregon have issued similar guidelines promoting expanded screening [15, 16].

Beyond screening, clinicians and public health authorities need to develop new approaches to ensure the treatment of diagnosed persons. The current therapy recommended by the Centers for Disease Control and Prevention for late latent syphilis or syphilis of unknown duration is 3 intramuscular injections of benzathine penicillin at weekly intervals. However, administering these to patients who are unstably housed and struggling with substance use disorders is extremely challenging. Successfully treating such patients will require new investments and innovations, including expanded outreach and treatment outside of clinical settings, greater use of doxycycline, creation of a clinical infrastructure that is more accessible to patients, and integration of clinical and social supports services.

In conclusion, King County is now experiencing an accelerating epidemic of syphilis among socially marginalized cisgender women and MSW accompanied by a dramatic increase in cases in among pregnant persons and cases of congenital syphilis. Our data suggest that there is a growing reservoir of undiagnosed infectious syphilis in these populations and an uncontrolled epidemic. Given increases in syphilis nationally, there is an urgent need to vastly expand syphilis screening beyond traditional clinical settings, especially among persons who use opioids or methamphetamine, persons who are unstably housed, and persons engaged in exchange sex. Expanded screening will need to be accompanied by new efforts to ensure patient treatment.

## Supplementary Material

ofad481_Supplementary_Data

## Supplementary Data

Supplementary materials are available at *Open Forum Infectious Diseases* online. Consisting of data provided by the authors to benefit the reader, the posted materials are not copyedited and are the sole responsibility of the authors, so questions or comments should be addressed to the corresponding author.

## Notes

***Author contributions.*** A.B. completed the data analysis for this article. All authors interpreted the data and provided critical review of the manuscript.
