# Supplementary material for: The Syphilis Epidemic Among Heterosexuals Is Accelerating: Evidence From King County, Washington
Source: Open Forum Infect Dis. 2023 Sep 23;10(10):ofad481. doi: 10.1093/ofid/ofad481 (PMC10578507; doi:10.1093/ofid/ofad481)
Supplement: ofad481_Supplementary_Data [file ofad481_supplementary_data.docx]

**Supplementary Figure 1: Pregnant syphilis among cisgender women by stage (bars) and congenital syphilis cases (line), King County 2007-2022**

**Supplementary Figure 2: Trends in the incidence of early and late latent/unknown duration syphilis among men who have sex with women (MSW), King County, Washington 2007-2022**

**Supplementary Figure 3: Joinpoint regression analysis of trends in the rate of all-stage syphilis among cisgender men who have sex with women (MSW) in King County, WA, 2007-2022**

**Supplementary Figure 4: Trends in the incidence of early and late latent/unknown duration syphilis among men who have sex with men (MSM), King County, Washington 2007-2022**

**Supplementary Figure 5: Joinpoint regression analysis of trends in the rate of all-stage, early, and late/unknown duration syphilis among cisgender men who have sex with men (MSM) in King County, WA, 2007-2022**
